# Dyadic Dynamics: The Impact of Emotional Responses to Facial Expressions on the Perception of Power

**DOI:** 10.3389/fpsyg.2018.01993

**Published:** 2018-10-25

**Authors:** Shlomo Hareli, Mano Halhal, Ursula Hess

**Affiliations:** ^1^The Laboratory for the Study of Social Perception of Emotions, University of Haifa, Haifa, Israel; ^2^Department of Business Administration, University of Haifa, Haifa, Israel; ^3^Department of Psychology, Humboldt University, Berlin, Germany

**Keywords:** dynamic expression of emotions, emotional interaction, reactive emotions, social power, anger, sadness

## Abstract

Emotion expressions play a central role in social communication, which, by definition is a dynamic process. Social communication involves the exchange of signals with temporal dynamic properties between two or more individuals. Nonetheless, emotion perception research has strongly focused on the study of single, static, unidirectional images. The goal of this research is to illustrate the dynamic nature of emotion communication by showing how the back and forth of a dyadic emotional interaction affects its perception by uninvolved observers. To that aim, we conducted three studies that investigated how observer’s inferences of social power are influenced by an exchange of emotions between members of a dyad. In Study 1, participants saw one person showing either anger or sadness to which the second member of the dyad reacted by showing either anger, fear or neutrality. In Study 1, only still photos were shown in sequence. In Studies 2 and 3, more dynamic stimuli and other emotions were included. Even though an angry expresser was always perceived as more powerful than a sad expresser, the emotional reactions of the interaction partner modulated perceived power. Across all three studies and different levels of dynamic stimuli, fear reactions always increased perceived power. Happiness, contempt and neutrality affected perceived power more selectively. This effect was mediated by the extent to which participants felt that the reaction of the second interaction partner suggested that the second interaction partner agreed with regard to the power differential between the two. Taken together, these experiments show that the social signal value of emotion expressions changes meaningfully as a function of the emotional response of the expressions’ target. Thus, the social signal value of emotions does not stand alone but has to be understood in the fuller context of the interaction.

## Introduction

Emotion expressions serve a social communicative function (Darwin, 1872/1965; Eibl-Ebesfeldt, 1989; Ekman, 1992; Fridlund, 1994; Hess et al., 1995; Fischer and Manstead, 2008; Shariff and Tracy, 2011; Scarantino, 2017) and most social interactions include exchanges of emotional expressions between the people involved (Frijda and Mesquita, 1994; Keltner and Haidt, 1999; Hareli and Rafaeli, 2008; Van Kleef, 2009). The study of the communication of emotions aims to understand how emotional signals are perceived and used by observers. This research focuses both on observers’ recognition of such expressions and the inferences about the expressers and the situation that they draw based on these expressions (Ekman et al., 1972; Hess et al., 2008; Van Kleef, 2010; Hareli, 2014).

However, the extant research is limited in two important respects. First, much of this research is restricted to the study of how a single, static, unidirectional expression of emotion is perceived (Krumhuber et al., 2013). Yet, social communication is by definition a dynamic process that involves an exchange of expressions between interaction partners (Hareli and Rafaeli, 2008). That is, the expressions shown by one interaction partner elicit expressions by the receiver. These can take different forms. Thus, the receiver may mimic the expression shown (Hess et al., 1999; Hess and Blairy, 2001; Hess and Fischer, 2013). Alternatively, the emotion shown by one interaction partner may elicit a reactive emotion in the other, which is then expressed by the addressee of the first expression (Hess and Fischer, 2013; Fischer and Hess, 2017). This latter response by the addressee of an emotion is an integral, but so far neglected, aspect of the emotion communication process.

Another limitation is the use of static images that are often bereft of context. This approach neglects informative aspects of expressive signals (see e.g., Ambadar et al., 2005; Krumhuber et al., 2013). Temporal characteristics only evident in dynamic displays impact both on the labeling of expressions and on the inferences about the expresser drawn from them (Krumhuber et al., 2013; Hess et al., 2016a).

The present research focuses on both of these points. Specifically, three studies explored how expressions of anger and sadness affect the attribution of social power as a function of the emotional response of the addressee of these emotions (i.e., reactive emotions). For this, participants saw not only the emotional expression of the person to be judged but also the emotional response of the addressee of this person’s expression. In addition, we assessed the impact of the temporal dynamics of the expressions by both parties. This goal was achieved by employing a strategy in which the complexity of the dynamic context was gradually increased across three studies. Study 1 used the frozen dynamism approach (Hareli et al., 2016), in which a timed sequence of still photos is shown to simulate an exchange of emotions between members of a dyad. This approach focusses participants’ attention on the different stages of the interaction. This enabled us to study, first, the effect of a mere exchange of expressive signals in an interaction. Study 2 went one step further by replacing the still photos of emotion expressions with videos. This allowed us to study whether dynamic expressions, which more closely resemble real life expressions, lead to the same effects in a social interaction. Finally, Study 3 used a video depicting the unfolding of an interaction involving the exchange of emotions between two persons appearing together. In all studies, a control condition in which only the expression of the person who is the focus of the judgment was included without any interactive context. Also, we tested a possible mechanism responsible for the combined effect of the emotions exchanged between the parties to the interaction. Overall, this research contributes to the understanding of how the dynamics of the social communication of emotions affects attributions of social power. In addition, it offers a research strategy allowing for a controlled examination of the social perception of dynamic interactions involving emotional exchanges between interaction partners.

In what follows, we will discuss how emotion expressions lead to inferences regarding an expresser’s social power and how reactive emotions are expected to affect such perceptions. This will serve as the basis for the specific hypotheses tested in this research.

Social power is a fundamentally important social factor (Russell, 1938), because it reflects a person’s ability to control others (Keltner et al., 2003). One cue to social power are emotion expressions (Keltner et al., 2003; Hareli and Rafaeli, 2008). Anger and sadness are among the most studied emotions in this context. Specifically, anger signals high social power and related constructs such as dominance (Keltner, 1995; Knutson, 1996; Averill, 1997; Hareli et al., 2009; Tiedens et al., 2016). Based on appraisal theory, anger signals high social power because it is associated with an appraisal that the expresser is able to control the environment (Keltner et al., 2003; Lerner and Tiedens, 2006). By contrast, expressions of sadness reflect low levels of social power as they are associated with appraisals of lack of control (Tiedens, 2001). Accordingly, anger expressions can be considered to be signals of high social power and expressions of sadness to be signals of a lack of power. This notion is in line with the assumption that emotion expressions communicate the expressers’ viewpoint in the situation (Dawkins and Krebs, 1978; Hess et al., 1995; Hareli and David, 2017; Scarantino, 2017).

Targets of such signals may respond with an expression of their own. Such responses are termed *reactive emotions* (Hess and Fischer, 2013). Reactive emotions are a direct response to the expression that elicited them. For example, if someone laughs in amusement and another person laughs as well, this can be seen as agreement that something funny happened. By contrast, if someone laughs and the other person looks irritated, this may suggest a fax pas.

In the present context, we focus on facial expressions that regulate the relationship between interaction partners. That is, we create a situation where the expressions of the interaction partners refer to each other. In that context, an anger expression, for example, signals that the expresser has control over the situation, and more specifically, control over the interaction partner (Keltner et al., 2003). In fact, the expression suggests that the interaction partner should conform to the angry person’s wishes. The reactive emotion of the addressed interaction partner then signals their perception of the power differential. Thus, if the other person shows a submissive emotion such as fear or sadness, they signal that the first person has more power than they have. By contrast, a dominant expression such as anger, contempt but also happiness (Knutson, 1996) should signal that they do not agree that the other person has more power than they do. The same rationale works for emotions signaling lack of social power such as sadness. If the second person shows fear in response to sadness, they signal that in their view, the other person, even though s/he does not signal much power, still has more power than they do. And, conversely, if the second person shows a dominant emotion, they signal that they also think that they have more power than the other person. That is, both emotion expressions “comment” on the power relationship within the dyad. These comments may agree or disagree with each other.

Importantly, for the observer, the second interaction partner is a second source of information. It makes sense for the observer to assume that this interaction partner has additional information about the sender and the situation and therefore can evaluate the relative power of the sender. As such, it makes only sense to prevail oneself of this additional information.

This implies that in a social interaction, anger or sadness expressions are not an absolute signal of power or the lack thereof simply because power is not an absolute attribute. The social power of any person depends on who else is present and therefore on the reactions of the addressee of these expressions. Also, we do not suggest that reactive emotions can completely change the perception of the initial emotions. As regards anger, since it is a signal of high social power, ignoring such a signal involves risks since even if a second person may think that they are at least equal in power, the first person may still have more power than the observer. Expressions of sadness, by contrast, reflect the admission of low social power. Since low power is socially undesirable, it is less likely to be attributed to ulterior motives and hence is likely to be trusted (Robinson et al., 1995). As such, there are (different) reasons for both emotions to be taken seriously. This is why reactive emotions are expected to modulate but not fundamentally change the meaning of anger and sadness for attribution of social power. This does, however, not mean that in real life interactions, where relative power and status are more relevant than absolute power and status, reactive emotions may not play a decisive role.

Hareli and David (2017) provided first evidence for such modulation. Specifically, they found that a person showing anger was perceived as having more social power when this anger was responded to with fear or sadness than when it was responded to with neutrality or anger. Further, this research also showed that the degree to which the expression of the second person was perceived by participants as congruent with the notion that the first person has more power than the second person mediated the effect of reactive emotions on perceived social power. Overall, they concluded that the perceived social power of the expresser is determined by the emotion shown and modified but not reversed by the reactive emotions of the interaction partner. While this research underscored the important role that reactive emotions play in social perception of emotions, several questions were left open.

First, Hareli and David (2017) did not include a no interaction control condition. That is, they could only compare the effect of different types of reactive emotions but could not assess the relevance of the absence or presence of a reaction. Second, as noted above, emotion expressions themselves are dynamic. Accordingly, it is important to understand whether the dynamics of the expressions exchanged between the interaction partners affect the attribution of power. The present research therefore addressed three questions. First, are attributions of social power to a person whose initial expression was reacted to by someone else, different from attributions of the same initial expression when shown alone? The latter situation reflects the typical paradigm used in this line of research. Second, we assessed the impact of expressive dynamics on this process.

Finally, we assessed the specific effect of different emotions. In particular, we compared the effect of fear reactions – which had previously been shown to increase perceptions of power (Hareli and David, 2017) to expressions of happiness (Study 1–3), contempt (Study 2) and anger (Study 3), as well as neutrality (Study 1 and 2). We predicted that emotions that signal high dominance (anger and happiness, Knutson, 1996) and emotions that suggest a devaluation of the expresser (contempt, Fischer and Roseman, 2007) would reduce perceptions of power. Also, specifically, they would reduce the degree to which the second person’s reaction is seen as a sign of acceptance of the high power signaled by the first person’s anger or conversely of the low power signaled by the first person’s sadness. Happiness also is an emotion that signals that the expresser considers that all is well (Scherer, 1987) thus in the present context this emotion may also be seen as mocking or denying especially expressions of anger signaling high power.

## Study 1

In Study 1 participants first saw one person expressing anger or sadness and then another person responding to this reaction with either fear, happiness or neutrality. In addition, in a control condition, participants saw only the first expresser. This enabled us to study the impact of the same expressions witnessed in isolation when they are not part of an unfolding social interaction.

### Methods

#### Participants

A total of 915 (477 men, 2 other) participants with a mean age of 38 years (*SD* = 11.5) who were recruited through Amazon MTurk, completed the study. Data collection continued with random assignment until a minimum of 25 participants per experimental cell was reached.

#### Materials and Procedure

Participants were randomly assigned to the social interaction or no social interaction condition. Participants in the social interaction condition were informed that they will see a series of photos taken from videos of an interaction between two persons, depicting a sequence of events in the interaction. The first photo was described as showing an expression by one interaction partner and the second the interaction partner’s response to this expression. No information about the nature of relationship between the two was provided. We assumed that in many situations this information is unknown to observers, although they may have guesses.

Participants in the no social interaction condition were informed that they will see a photo of a person. All participants were told that they will have to rate different things about what they saw. Each participant completed only one trial.

**FIGURE 1:**
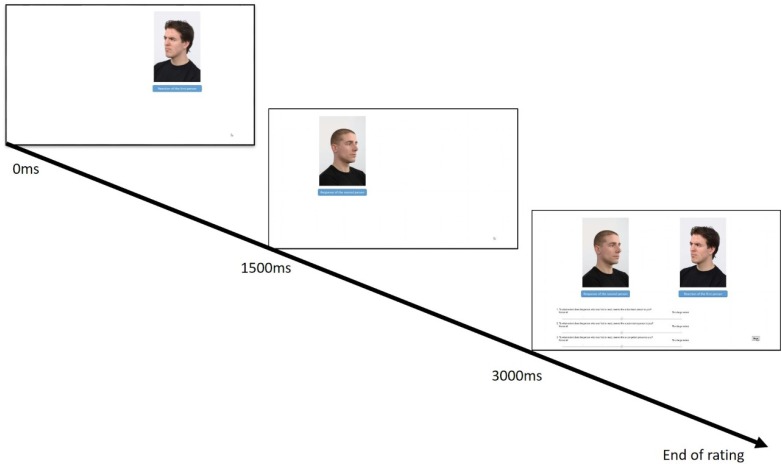
Sequence of stimulus presentation and set-up with example of stimuli used in Study 1.

As posers we randomly chose 8 men and 8 women from the Radboud Faces Database (Langner et al., 2010). Of these, four posers from each gender showing either anger or sadness, served as the first expresser in the social interaction condition or the only expresser in the no social interaction condition. The remaining 4 posers of each gender expressing fear, happiness and neutrality, served as the second expresser. Dyads were formed by randomly selecting one poser from the set of first expressers and one from the set of second expressers. To increase the impression that reactions were taken from actual interactions, we used the 45° left and right orientations versions of the photographs, so that the expressers appeared to orient their reactions toward one another. To control for the effect of orientation and side of presentation, half of the participants saw the sets with first expresser person appearing on the right hand side of the screen, orienting the expression toward the left, and the person reacting to this expression appearing on the left and orienting the reaction toward the right. The rest of the participants saw the sets with the reversed position of expressers and orientations. To further establish the impression that the stimuli represent a sequence of reactions, the photograph depicting the person expressing the emotion first appeared for 1,500 ms after which it disappeared, and the person reacting to this expression then appeared on the other side for 1,500 ms. Below the photographs was written: “The reaction of the first person” and “The response of the second person,” for the first and second photos, respectively (for an example of a stimulus and the sequence of events, see Figure 1). Next, both photographs were presented in their original position and rating scales appeared below.

In the no social interaction condition, a poser from the first expresser set was selected. This poser appeared either in the right gaze or left gaze orientation in the respective position as the first poser in the social interaction position. The photo appeared first for 1,500 ms and then disappeared. Then the photo reappeared together with the rating scales. No inscription appeared under the photo in this condition.

#### Dependent Measures

Participants were asked to rate their perception of the first expresser’s dominance, submissiveness and competence as well the expresser’s control over the situation. Since these measures correlated highly (α = 0.76; ω = 0.85^1^), they were combined into one social power scale by computing the average of these ratings with submissiveness being reverse scored. Then participants were asked to rate the intensity of anger and sadness of the person who was shown first (or the only person shown, for the no social interaction condition). We further assessed to what degree participants considered the expression of the second person to signal that they accepted the first expresser’s dominance, submitted to the first expresser and confirmed the first expresser’s standing in the interaction. These measures correlated (*α* = 0.82, ω = 0.89) and hence were combined by averaging the ratings into one scale, which we labeled “acceptance of power.” All ratings were made on 7-point Likert scales anchored with 1 = not at all and 7 = to a large extent.

## Results

### Emotion Perception

#### Emotions of first expresser

Initial analyses did not reveal any significant main effects nor interactions involving sex of either interaction partner for anger ratings for the first expression. A significant effect on sadness ratings for the first expression did not yield any significant *post hoc* effects. The two gender factors were therefore dropped from further analyses. A 2 (emotion shown by the first person: sadness, anger) × 4 (emotion shown by the second person: no emotion, neutral, fear, happiness) analysis of variance on the emotion ratings yielded for anger, *F*(1,907) = 829.83, *p* < 0.001, $\eta_{p}^{2}$ = 0.48, a main effect of first emotion such that anger expressions were rated as showing more anger (*M* = 5.43, *SD* = 1.56, CI: 5.29, 5.58) than sadness expressions (*M* = 2.46, *SD* = 1.55, CI: 2.32, 2.61). For sadness, a main effect of first emotion emerged, *F*(1,907) = 992.85, *p* < 0.001, $\eta_{p}^{2}$ = 0.52, as well, such that sadness expressions were rated as sadder (*M* = 5.96, *SD* = 1.30, CI: 5.82, 6.10) than anger expressions (*M* = 2.78, *SD* = 1.73, CI: 2.64, 2.92). In addition, for sadness only, a main effect of second emotion emerged, *F*(3,907) = 3.08, *p* = 0.027, $\eta_{p}^{2}$ = 0.01, such that overall, across both emotion conditions, expressions that were reacted to with fear were rated as less sad (*M* = 4.17, *SD* = 2.24, CI: 3.93, 4.33) than those that were shown alone (*M* = 4.48, *SD* = 2.18, CI: 4.37, 4.76). Expressions reacted to with happiness (*M* = 4.40, *SD* = 2.23, CI: 4.21, 4.61) and with a neutral expression (*M* = 4.38, *SD* = 2.18, CI: 4.17, 4.57) were not rated differently from one another. The interaction effect was not significant, *F*(3,907) = 0.30, *p* = 0.826, $\eta_{p}^{2}$ = 0.00. Thus, overall, the emotions were interpreted as intended. It is interesting to note that a fear reaction by the addressee of either an anger or sad expression makes this expression appear sadder. The absence of an interaction effect suggests that this may be more of a halo effect.

#### Perceived social power of the first expresser

Initial analyses did not reveal any significant main effects nor interactions involving sex of either interaction partner for perceived power or perceived acceptance of power by the second person. The two gender factors were therefore dropped from further analyses. A 2 (emotion shown by the first person: sadness, anger) × 4 (emotion shown by the second person: no emotion, neutral, fear, happiness) analysis of variance was conducted on the attribution of social power. A significant main effect of first expression, *F*(1,907) = 358.11, *p* < 0.001, $\eta_{p}^{2}$ = 0.28, emerged, such that individuals who showed anger were rated as higher in social power (*M* = 4.60, *SD* = 1.14, CI: 4.51, 4.72) than those who showed sadness (*M* = 3.22, *SD* = 1.16, CI: 3.12, 3.32). Further, a significant main effect of second expression, *F*(3,907) = 21.30, *p* < 0.001, $\eta_{p}^{2}$ = 0.07, emerged. *Post hoc* analyses revealed that any expression reacted to with fear resulted in higher attributions of social power (*M* = 4.41, *SD* = 1.34, CI: 4.28, 4.57) than expressions reacted to with happiness (*M* = 3.70, *SD* = 1.32, CI: 3.55, 3.84) or neutrality (*M* = 3.78, *SD* = 1.34, CI: 3.64, 3.93), or not responded to at all (*M* = 3.79, *SD* = 1.26, CI: 3.62, 3.90) which did not differ. The interaction was not significant, *F*(3,907) = 0.26, *p* = 0.857, $\eta_{p}^{2}$ = 0.00. That is, contrary to expectations, the effect of the reactive emotion did not depend on the first emotion shown. Thus, being responded to with fear increased perceived social power regardless of whether high or low social power were signaled.

#### Perceived acceptance of power by the second expresser

A 2 (emotion shown by the first person: sadness, anger) × 3 (emotion shown by the second person: neutral, fear, happiness) analysis of variance was conducted on the degree to which participants considered that the second person accepted that the first person has more power. A significant main effect of first expression, *F*(1,671) = 44.39, *p* < 0.001, $\eta_{p}^{2}$ = 0.06, and of second expression, *F*(2,671) = 72.84, *p* < 0.001, $\eta_{p}^{2}$ = 0.18, emerged. This main effect was qualified by an interaction *F*(2,671) = 9.11, *p* < 0.001, $\eta_{p}^{2}$ = 0.03, such that when anger was shown first, acceptance of power was perceived as strongest when the reactive emotion was fear (*M* = 4.78, *SD* = 1.40, CI: 4.50, 5.05), followed by neutrality (*M* = 3.61, *SD* = 1.59, CI: 3.34, 3.88), and least for happiness (*M* = 2.62, *SD* = 1.52, CI: 2.35, 2.89). The same pattern was found for sadness: fear (*M* = 3.64, *SD* = 1.45, CI: 3.37, 3.91), then neutrality (*M* = 2.58, *SD* = 1.37, CI: 2.31, 2.85) and happiness (*M* = 2.54, *SD* = 1.43, CI: 2.27, 2.81), yet, neutrality and happiness did not differ significantly. Thus, independent of whether the first expression was anger or sadness, participants saw fear as a sign that the second expresser considered the first to be high(er) in power, and neutrality and happiness as doing so to a much lesser degree. This is congruent with the finding reported above that fear reactions always increased the perceived power of the first expresser. We therefore conducted a mediation analysis to assess whether this increase in perceived power is due to the fact that the expression was seen as supportive of the notion that the first expresser is high(er) in power.

### Mediation Analysis

To analyze the proposed mediation, we calculated a mediation model (Hayes model 4) with reactive emotion as a multicategorial index coded variable comparing fear and happiness to neutral. The analysis used Process 3.0 (Hayes, 2017).

A significant positive indirect effect on perceived social power for reactive fear expressions (*b* = 0.41, *SE* = 0.06, CI: 0.29, 0.54) and a significant indirect effect for happiness (*b* = -0.19, *SE* = 0.06, CI: -0.30, -0.08) compared to neutral emerged. Specifically, reactive fear expressions were rated as signaling acceptance of the first person’s power by the second person and this acceptance in turn increased attributions of social power to the first person by the participants. The converse effect was found for happiness reactions (even though this effect did not yield a significant effect in the ANOVA). Thus, as predicted, the emotional expression of the addressee of an expression impacts on the inferences that observers draw about the sender of that expression because these expressions themselves speak meaningfully toward the social power of the first person.

## Discussion

Overall, the present findings replicate and extend findings by Hareli and David (2017). We found again that a fear reaction by the addressee of an expression leads to attributions of higher social power to the person sending the initial expression. In Study 1, this was independent of whether the initial expression was anger or sadness.

We further found that this increase in attributed social power was mediated by the fact that anyone who is reacted to with fear is seen as more powerful than someone who is reacted to with neutrality. Interestingly, the converse was found for happiness in the mediation analysis. That is, anyone who was reacted to with happiness was rated as lower in social power to the degree that this expression seemed to dispute claims of social power. This finding is suggestive of the notion that reactions of happiness may contradict signals of high social power.

## Study 2

Even though the findings of Study 1 support our basic hypotheses that the emotional reactions of both partners in an interaction are relevant for observers’ social judgments, the setting we used was somewhat artificial. Participants saw two still photos of individuals supposedly interacting rather than actual dynamic expressions. Thus, in Study 2, using the same methodology as in Study 1, the still photos were replaced by videos of expressions of emotions with the goal of examining to what degree the findings of Study 1 replicate in such conditions.

In addition, we added expressions of contempt as an additional reactive emotion as one goal of the present research was to examine if and under what conditions a reactive emotion can decrease the perceived social power of the first expresser. Contempt is considered a response that devalues its objects to the point of nullifying them and their capabilities (Fischer and Roseman, 2007). Thus, a contempt reaction by the addressee of a “power claim” by the first expresser should undermine this claim.

In Study 2 we also measured the perceived intensity of reactive emotions. We did this because ratings of perceived emotions more accurately reflect the participants’ perception of these expressions than do the categorical condition codes. Finally, since we did not find significant effects for gender composition in Study 1, we simplified the design by using same-sex dyads only.

### Methods

#### Participants

A total of 593 (343 women, 1 other) participants with a mean age of 40 years (*SD* = 12.6) who were recruited through Amazon MTurk completed the study. Data collection continued with random assignment until a minimum of 25 participants per experimental cell was reached.

#### Materials and Procedure

The procedure was the same as in Study 1 except for the fact that videos were used as the primary stimuli. As posers we randomly chose 4 men and 4 women from the Amsterdam Dynamic Facial expressions Set (Van der Schalk et al., 2011). To increase the impression that reactions were taken from actual interactions, we used the 45° turning right versions from the set. To control for the effect of orientation and side of presentation, videos were rotated 180° using video editing software (Camtasia Studio 8, Techsmith^2^). Thus, as in Study 1, the orientation of the first expresser was counterbalanced. Videos were edited to start with the expresser showing a neutral expression. Emotion expressions started after 500 ms. and the reaction unfolded and lasted for an additional 5000 ms. Combination of expressers was random with the restrictions that the two posers were different actors of the same sex. As in Study 1, the first expresser appeared first and the video with the person reacting to this expression then appeared on the other side after the end of the first video. Next, photographs created from the apex of the reaction in the video were presented in their original position and rating scales appeared below. Below the videos and photographs it was written: “The reaction of the first person” and “The response of the second person,” for the first and second stimuli, respectively. In the no social interaction condition, only one poser appeared either in the right gaze or left gaze orientation. When the video was finished, the video and photo of the apex of the reaction appeared with the rating scales. No inscription appeared under the video and photo in this condition. This resulted in a 2 (Emotion of first expresser: anger or sadness) × 2 (Gender of the expressers) × 5 (Reactive emotion of second expresser: fear, contempt, happiness and neutrality, no reaction) between-subjects design.

#### Dependent Measures

The same dependent measures as in Study 1 were used. Ratings of perceived dominance, submissiveness, competence and control over the situation were combined into one social power scale (α = 0.73, ω = 0.82). The ratings of the extent to which the person who was second to express an emotion submitted to the first expresser, accepted the first expresser’s dominance and confirmed the first expresser’s standing in the interaction were combined into one acceptance of social power scale (α = 0.68, ω = 0.64). For self-report questionnaire items, internal consistencies of 0.70 are often considered acceptable if scales consist of very few items (Hahn et al., 2012), as is the case here.

Participants further rated the perceived intensity of anger and sadness of the person who was shown first (or the only person shown, for the no social interaction condition) as well as perceived intensity of the reactive emotions of fear, contempt, happiness and neutrality in the social interaction condition. All ratings were made on 7-point Likert scales anchored with 1 = not at all and 7 = to a large extent.

## Results

### Emotion Perception

#### Emotions of first expresser

A 2 (First expression) × 2 (Gender of expressers) × 5 (Reactive emotion) ANOVA was conducted on ratings of anger and sadness intensity. For ratings of anger, a significant main effect of first expression emerged, *F*(1,573) = 573.46, *p <* 0.001, $\eta_{p}^{2}$ = 0.50, such that expressions of anger were rated as angrier (*M* = 5.78, *SD* = 1.40, CI: 5.60, 5.95) than expressions of sadness (*M* = 2.76, *SD* = 1.66, CI: 2.60, 2.95). The main effect of reactive emotion was also significant, *F*(4,573) = 2.69, *p* = 0.03, $\eta_{p}^{2}$ = 0.02. *Post hoc* tests revealed that anger intensity was rated somewhat lower when it was responded to by contempt (*M* = 4.00, *SD* = 2.22, CI: 3.75, 4.28) or neutrality (*M* = 4.19, *SD* = 2.16, CI: 3.85, 4.40) compared to when shown alone (*M* = 4.65, *SD* = 2.08, CI: 4.34, 4.90). When anger was responded to by fear (*M* = 4.22, *SD* = 2.15, CI: 4.02, 4.58) or by happiness (*M* = 4.26, *SD* = 2.13, CI: 4.04, 4.59) perceived intensity of anger did not differ from any other condition.

For ratings of sadness, a significant main effect of first expression emerged, *F*(1,573) = 502.21, *p <* 0.001, $\eta_{p}^{2}$ = 0.47, such that expressions of sadness were rated as sadder (*M* = 5.70, *SD* = 1.69, CI: 5.52, 5.90) than expressions of anger (*M* = 2.64, *SD* = 1.67, CI: 2.46, 2.84). In addition, a significant gender by first emotion interaction emerged, *F*(1,573) = 13.10, *p <* 0.001, $\eta_{p}^{2}$ = 0.02. *Post hoc* tests indicated that women’s sadness was perceived as somewhat more intense (*M* = 5.99, *SD* = 1.42, CI: 5.71, 6.25) than men’s sadness (*M* = 5.44, *SD* = 1.88, CI: 5.17, 5.70) and men’s anger was rated as somewhat sadder (*M* = 2.87, *SD* = 1.75, CI: 2.60, 3.14) than women’s anger (*M* = 2.43, *SD* = 1.55, CI: 2.17, 2.70). Overall, these results indicate that the emotions of the first expresser were perceived as planned.

#### Perceived intensity of reactive emotions

A 2 (First expression) × 2 (Gender of expressers) × 4 (Reactive emotion) ANOVA was conducted on ratings of fear, contempt, happiness and neutrality. A main effect of reactive emotion emerged for all emotions (see Table 1). Ratings on each of the four emotion scales were highest for the video with the corresponding focal emotion expression. However, additional effects emerged for secondary emotion ratings. That is, for emotions not actually expressed, for example, perceived fear of a face showing anger. Contempt expressions were rated as more neutral than fear and happiness expressions and fear expressions were rated as less contemptful than happiness and neutral expressions. Contempt expressions were rated as happier than expressions of fear and neutrality.

**Table 1:** Ratings of perceived intensity of reactive emotions as a function of expressed reactive emotion – Study 2 and Study 3.

Reactive emotion	Fear			Happiness			Contempt			Neutrality			*F* (3,464)	*p*	$\eta_{p}^{2}$
							
Study 2	*M*	*SD*	95% CI	*M*	*SD*	95% CI	*M*	*SD*	95% CI	*M*	*SD*	95% CI			
Fear	5.74_a_	1.58	5.51, 6.06	1.67_b_	1.36	1.40, 1.94	2.04_c_	1.47	1.78, 2.31	2.63_d_	1.80	2.33, 2.31	180.86	<0.001	0.54
Happiness	1.74_a_	1.36	1.50, 1.99	6.17_b_	1.11	5.94, 6.42	2.38_c_	1.65	2.14, 2.61	1.74_a_	1.10	1.52, 2.01	305.79	<0.001	0.66
Contempt	2.60_a_	1.64	2.27, 2.93	3.41_b_	2.08	3.07, 3.73	4.46_c_	1.81	4.14, 4.78	3.79_b_	1.81	3.46, 4.13	21.82	<0.001	0.12
Neutrality	1.68_a_	1.27	1.38, 1.95	1.67_a_	1.10	1.39, 1.95	3.33_b_	1.86	3.06, 3.60	5.21_c_	1.79	4.93, 5.50	140.29	<0.001	0.48

**Study 3**	**Fear**			**Happiness**			**Anger**						**(2, 334)**		

Fear	4.62_a_	1.84	4.29, 4.90	1.99_b_	1.42	1.67, 2.28	3.22_c_	1.72	4.29, 4.90				71.35	<0.001	0.30
Happiness	1.99_a_	1.36	1.70, 2.28	5.44_b_	1.81	5.17, 5.74	2.09_a_	1.40	1.78, 2.35				185.93	<0.001	0.53
Anger	2.51_a_	1.64	2.24, 2.83	2.09_a_	1.57	1.79, 2.39	4.84_b_	1.68	4.54, 5.13				94.72	<0.001	0.36
Neutrality	2.06	1.55	1.79, 2.36	2.21	1.62	1.91, 2.48	2.32	1.55	2.02, 2.60				0.65	=0.52	0.00


*Means with different subscripts differ at *p* < 0.05. In Study 3 there was no condition of a reactive emotion of neutrality but participants were asked to rate each expression on perceived neutrality.*

For fear ratings, a significant main effect of first emotion emerged, *F*(1,464) = 21.86, *p <* 0.001, $\eta_{p}^{2}$ = 0.05, such that fear was rated somewhat more intensely when it was expressed in response to anger (*M* = 3.30, *SD* = 2.26, CI: 3.16, 3.54) than in response to sadness (*M* = 2.69, *SD* = 2.16, CI: 2.51, 2.89). For neutrality ratings, a main effect of expresser gender emerged, *F*(1,464) = 4.41, *p* = 0.04, $\eta_{p}^{2}$ = 0.009, such that men were rated as somewhat more neutral overall (*M* = 3.08, *SD* = 2.18, CI: 2.92, 3.31) than women (*M* = 2.85, *SD* = 2.04, CI: 2.62, 3.02). Thus, overall, reactive emotions were perceived as planned.

#### Perceived social power of the first expresser

We first compared the effect of a reactive emotion on the evaluation of the expression alone. For this a 2 (First expression) × 2 (Gender of expressers) × 4 (Reactive emotion) ANOVA was conducted on ratings of social power. A significant main effect of first expression emerged, *F*(1,573) = 266.05, *p* < 0.001, $\eta_{p}^{2}$ = 0.32, such that individuals showing anger expressions (*M* = 4.53, *SD* = 1.13, CI: 4.41, 4.65) were rated as higher in social power than those who showed sadness (*M* = 3.10, *SD* = 1.10, CI: 2.98, 3.23). A first expression × gender interaction was significant, *F*(1,573) = 4.20, *p* = 0.041, $\eta_{p}^{2}$ = 0.01, but *post hoc* tests did not reveal significant differences as a function of gender. Further, as in Study 1, the main effect of second expression was significant, *F*(4,573) = 13.93, *p* < 0.001, $\eta_{p}^{2}$ = 0.09, but in Study 2 also qualified by a first expression by second expression interaction, *F*(4,573) = 3.30, *p* = 0.011, $\eta_{p}^{2}$ = 0.02. As shown in Table 2, for both sadness and anger expressions, as in Study 1, reactive fear expressions increased attributions of social power relative to the expression shown alone. In addition, for anger expressions, reactive happiness expressions reduced the attribution of social power relative to the expression shown alone. This effect of reactive happiness was hinted at in the mediation analysis for Study 1, but not significant when comparing means. No other significant differences emerged. In sum, for sad expressions only fear and for anger expressions both fear and happiness moderated the perception of social power compared to the expression alone.

**Table 2:** Perceived social power as a function of first expresser’s emotion and reactive emotion – Study 2 and Study 3.

	Reactive emotion									
	
Study 2	No Emotion		Fear		Happiness		Contempt		Neutral	
					
First emotion	*M SD*	95% CI	*M SD*	95% CI	*M SD*	95% CI	*M SD*	95% CI	*M SD*	95% CI
Sadness	3.03_a_ 0.99	2.75, 3.32	3.58_b_ 1.05	3.31, 3.84	3.01_a_ 1.21	2.74, 3.27	2.86_a_ 0.98	2.60, 3.13	3.04_a_ 1.15	2.76, 3.32
Anger	4.64_c_ 0.95	4.38, 4.92	5.25_d_ 0.85	4.97, 5.53	3.84_b_ 1.09	3.57, 4.11	4.50_c_ 1.16	4.24, 4.77	4.44_c_ 1.11	4.14, 4.68

**Study 3**	**No Emotion**		**Fear**		**Happiness**		**Anger**			

Sadness	2.84_a_ 0.90	2.57, 3.08	3.79_c_ 1.21	3.53, 4.05	3.57_c_ 1.19	3.27, 3.79	4.17_b_ 1.18	3.93, 4.43		
Anger	4.75_d_ 0.72	4.49, 5.00	5.18_e_ 0.79	4.93, 5.42	4.66_d_ 0.85	4.41, 4.90	4.86_de_ 0.86	4.61, 5.11		


*Means with different subscripts differ at *p* < 0.05.*

We then assessed the effects of reactive emotions as perceived by the participants. Specifically, it can be argued that the effect of the reactive emotions depends on the perceived emotion rather than emotion condition. Specifically, even if a face has been validated as showing anger, a given participant may also perceive secondary emotions such as sadness and fear. Secondary emotions have been shown to affect perceptions of interactions in meaningful ways (Hess et al., 2016b). In fact, as can be seen in Table 1 above, even though the focal emotion was rated as strongest for each of the expressions, participants perceived a mix of expressions as is common in emotion perception (Russell and Fehr, 1987; Russell et al., 1993; Yrizarry et al., 1998; Hess et al., 2016b). We therefore conducted multiple regression analyses with the emotion ratings for the reactive emotion as predictors. Given the first emotion by second emotion interaction reported above, we ran separate analyses for sad and anger first expressions. Given the weakness of the gender × first emotion effect, gender was dropped from this analysis.

For reactions to sadness, the MR model explained 12% of the variance, *F*(4,238) = 7.89, *p* < 0.001. Significant effects emerged for fear (β = 0.28, *p* < 0.001, CI: 0.15, 0.42) and contempt (β = -0.17, *p* = 0.007, CI: -0.30, 0.05). Specifically, whereas fear reactions to sadness increased perceptions of social power, contempt reactions to sadness reduced it. That is, contempt reduced the already weak signal of power shown by the first expresser.

For reactions to anger, the MR model explained 29% of the variance, *F*(4,232) = 23.80, *p* < 0.001. Significant effects emerged for fear (β = 0.33, *p* < 0.001, CI: 0.21, 0.45), contempt (β = -0.13, *p* = 0.027, CI: -0.24, -0.01) and happiness (β = -0.27, *p* < 0.001, CI: -0.39, -0.15). Again, whereas fear increased perceptions of social power both contempt and happiness decreased it.

#### Perceived acceptance of power by the second expresser

We then assessed to what degree the reactive emotions shown by the addressee of the first expressions were seen as accepting that the first person has more power. Congruent with the analyses above, we calculated MR separately for sadness and anger first expressions with reactive emotion ratings as predictors.

For reactions to sadness, the MR model explained 19% of the variance, *F*(4,238) = 13.84, *p* < 0.001. Only fear significantly and positively predicted the degree to which the expression of the second person signaled that they considered the first person to have (more) power (β = 0.42, *p* < 0.001, CI: 0.30, 0.55).

For reactions to anger, the MR model explained 40% of the variance, *F*(4,232) = 39.12, *p* < 0.001). Significant effects emerged for fear (β = 0.49, *p* < 0.001, CI: 0.38, 0.60), contempt (β = -0.19, *p* < 0.001, CI: -0.29, -0.09) and happiness (β = -0.15, *p* = 0.010, CI: -0.26, -0.04). Specifically, reactions of fear increased, whereas reactions of contempt and happiness decreased the degree to which the response by the addressee of an anger expression was considered supportive of the notion that the anger expresser had high(er) social power.

### Mediation Analysis

As for Study 1, we conducted mediation analyses to assess whether the increases and decreases in perceived social power as a function of reactive emotion can be explained by the degree to which these expressions were perceived as accepting that the first person has high(er) power. For this, we defined a saturated model in AMOS (22.0) in which the four emotion rating variables predicted the degree of acceptance of power and this variable in turn predicted perceived social power. We conducted the analyses separately for sadness and anger first expressions. Bootstrap was set to 3000.

For reactions to sadness, only for fear was the indirect effect significant (β = 0.12, *p* < 0.001, CI: 0.06, 0.20). For reactions to anger, significant indirect effect were found for fear (β = 0.24, *p* < 0.001, CI: 0.17, 0.33), contempt (β = -0.10, *p* = 0.002, CI: -0.16, -0.04) and happiness (β = -0.07, *p* = 0.012, CI: -0.14, -0.02).

## Discussion

In sum, the mediation analyses confirmed the notion that the effects of reactive emotions on perceived social power were mediated by the perception that the second expresser considered the first expresser to be high(er) in power. Specifically, fear reactions in response to both anger and sadness expressions increased perceived social power to the degree to which these reactions were seen as accepting the power signaled by the first person. For contempt and happiness expressions shown in reaction to anger, the converse effect was found. The effects for fear and happiness replicate findings from Study 1. The finding for contempt supports the notion that contempt can invalidate the power signaled by anger expressions. For sadness expressions, contempt also had the effect of eroding the already low level of power signaled by that expression even further. Yet, this was not mediated through the perception that this expression signals that the second perceiver disagrees with the power claim by the first perceiver. One possibility is that contempt shown toward a sad person may devalue the person as such (Fischer and Roseman, 2007) – rather than their “claim” and this may also lead to perceived lack of social power.

Overall, the results of Study 2 further support the notion that not only the expression shown by a person but also the reactions of others to this expression are relevant for the assessment of the social power of the individual. That is, in a dyadic interaction, the emotional expressions of both interaction partners meaningfully inform observers about the expressers. Interestingly, whereas in Study 1, the type of emotion shown by the first expresser did not affect the impact of the reactive emotions, it did so for Study 2. Specifically, as proposed in the introduction, reactions of happiness in response to anger but not in response to sadness had a power eroding effect. This, because showing happiness and signaling that all is well in the face of an aggressive signal such as anger suggests that the happy person does not consider the threat display threatening. Someone who smiles at a sad person, by contrast, might be seen as callous more than anything else and hence their display is disregarded for evaluations of the social power of the sad person.

Importantly, the replication of findings from Study 1, showed that the effects were not driven by the artificial nature of the stimulus display in that study. That stronger additional effects of happiness were found may be due to the use of dynamic rather than static images.

Yet, this study too is limited in two important respects. First, the emotion expressions were presented to the participants in sequence and then were shown as stills during the rating task. Even though this enables participants to focus carefully on the sequence of the events, it may also over sensitize them to aspects of the situation that otherwise may be more subtle. That is, when people witness a dyadic social interaction, both partners appear together and the focus of the observers may shift between the two, forcing them to be less aware of each individual expression. In addition, the mere presence of both partners together may provide important information about the interaction that is missing when the stimuli are presented sequentially. Further, the videos we used in Study 2 showed expressions that were quite intense. Real-life expressions of emotions are often considerably less intense (Motley and Camden, 1988).

## Study 3

Given the limitations of Study 2, as described above, the goal of Study 3 was to test our hypotheses using a more ecological valid design in which both interaction partners are showing more subtle facial expressions concurrently. Finally, the emotion ratings showed that contempt was notably less well recognized than fear and happiness. Since the recognition of contempt would likely be even further reduced for expressions with lower intensity, we replaced contempt in Study 3 with expressions of anger. Anger was also expected to serve as a signal that the addressee, especially of an anger expression, does not agree that the other is (more) powerful (Hareli and David, 2017).

### Methods

#### Participants

A total of 457 (274 women) participants with a mean age of 40 years (*SD* = 13.3) who were recruited through Amazon MTurk. Data collection continued with random assignment until a minimum of 25 participants per experimental cell was reached.

#### Materials and Procedure

**FIGURE 2:**
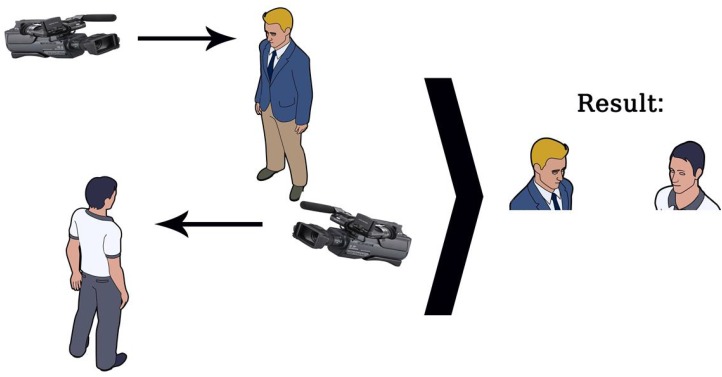
Diagram of supposed set-up of creating the videos of social interactions for Study 3 presented to participants in the social interaction condition as part of the instructions. The individual videos of each person were supposedly merged into one video as presented to participants.

The still photos that were created from the videos and were used in Study 2 as the stimuli for the second phase of the study, were used to create morphed videos with an expression changing from neutral to one of the expressions (anger, sadness, fear, or happiness) using Fantamorph 5.0 (Abrosoft)^3^. Morphed videos were saved as AVI video files. Videos ended when the expression reached 80% of their peak intensity along the continuum from a neutral expression to the apex of the emotion. For the conditions involving an interaction, videos of two posers of the same sex were placed, one next to the other, each orienting toward the other. The video of the first expresser was edited so that the expression started after 500 ms. The expression in the video of the responder started 1000 ms later. Both reactions reached their respective apex (80% of the original apex) after 1000 ms, respectively and the entire sequence lasted for 3000 ms. We provided the participants with the explanation that the video shows an expression by one interaction partner and how the other interaction partner responded to this expression and that each partner was filmed with a different camera. To clarify this fictitious set up supposedly creating the presented stimuli, a figure depicting how the scene was created was shown (in the social interaction condition only, see Figure 2). Participants were further told that the video would be shown twice so that they can have a better sense of what went on. As in Study 2, combination of expressers was random with the restrictions that both posers were different individuals and that they were of the same sex. Presentation orientation of the posers was counterbalanced, as in Studies 1 and 2. In the no social interaction condition, only one poser appeared either in the right gaze or left gaze orientation in the respective position as the first poser in the social interaction condition. Unlike in Studies 1 and 2, no inscription appeared under the videos in any condition. This resulted in a 2 (Emotion of first expresser: anger or sadness) × 2 (Gender of the expressers) × 4 (Reactive emotion of second expresser: no reactive emotion, fear, happiness, and anger) between-subjects design.

#### Dependent Measures

The same ratings and scales as in Study 2 were used, except that the contempt rating was replaced with an anger rating. As in the previous studies, measures of perceived dominance, submissiveness, competence and control over the situation were combined into one social power scale (α = 0.71, ω = 0.63) and measures of submission to the first expresser, acceptance of the first expresser’s dominance and confirmation of the first express’s standing in the interaction were combined into the acceptance of power scale (α = 0.61, ω = 0.79).

## Results

### Emotion Perception

#### Emotions of first expresser

A 2 (First expression) × 2 (Gender of expressers) × 4 (Reactive emotion) ANOVA was conducted on ratings of anger and sadness intensity. For ratings of anger, a significant main effect of first expression emerged, *F*(1,441) = 188.98, *p <* 0.001, $\eta_{p}^{2}$ = 0.30, such that expressions of anger were rated as angrier (*M* = 5.15, *SD* = 1.64, CI: 4.93, 5.35) than expressions of sadness (*M* = 3.07, *SD* = 1.65, CI: 2.84, 3.26). In addition, a significant interaction between first emotion and reactive emotion emerged, *F*(3,441) = 4.67, *p* = 0.003, $\eta_{p}^{2}$ = 0.03. *Post hoc* tests revealed that perceived anger intensity was always higher for anger expressions than for sadness expressions and all anger expressions were rated similarly irrespective of reactive emotion (*M* = 5.43, *SD* = 1.26, CI: 4.99, 5.84; *M* = 5.10, *SD* = 1.50, CI: 4.67, 5.52; *M* = 4.72, *SD* = 2.00, CI: 4.28, 5.10; and, *M* = 5.36, *SD* = 1.61, CI: 4.94, 5.76, for anger with no reaction, anger reacted to with anger, happiness, and fear, respectively). However, anger ratings of sadness expressions varied with reactive emotions. Specifically, sadness responded to with fear was rated as less angry (*M* = 2.42, *SD* = 1.49, CI: 1.98, 2.86) than sadness responded to with anger (*M* = 3.57, *SD* = 1.55, CI: 4.67, 5.52). No difference emerged between sadness shown alone (*M* = 3.13, *SD* = 1.74, CI: 3.14, 3.98) and sadness reacted to with happiness (*M* = 3.11, *SD* = 1.63, CI: 2.66, 3.52).

For ratings of sadness, a significant main effect of first expression, *F*(1,441) = 147.64, *p <* 0.001, $\eta_{p}^{2}$ = 0.25, emerged, such that expressions of sadness were rated as sadder (*M* = 4.73, *SD* = 2.00, CI: 4.51, 4.97) than expressions of anger (*M* = 2.78, *SD* = 1.57, CI: 2.55, 2.99). In addition, there was a significant main effect of reactive emotion, *F*(3,441) = 10.35, *p <* 0.001, $\eta_{p}^{2}$ = 0.07. *Post hoc* tests indicated that sadness was perceived as somewhat more intense when participants saw it alone (*M* = 4.50, *SD* = 1.97, CI: 4.19, 4.84) than in any other condition which did not differ (*M* = 3.59, *SD* = 1.80, CI: 3.28, 3.91; *M* = 3.59, *SD* = 2.18, CI: 3.31, 3.95; and *M* = 3.22, *SD* = 1.98, CI: 2.97, 3.61, for sadness – anger, sadness – happiness and, sadness – fear, respectively). Overall, these results indicate that the emotions of the first expresser were perceived as planned.

#### Perceived intensity of reactive emotions

A 2 (First expression) × 2 (Gender of expressers) × 3 (Reactive emotion) ANOVA was conducted on ratings of fear, happiness, anger and neutrality intensity. A main effect of reactive emotion emerged for all emotions, except for ratings of neutrality (see lower part of Table 1). For each emotion, as expected, ratings were highest on the scale that corresponded to the focal emotion for that expression.

For ratings of neutrality, the only effect that emerged was a main effect of gender, *F*(1,334) = 8.08, *p* = 0.005, $\eta_{p}^{2}$ = 0.02, indicating that men were rated as somewhat more neutral (*M* = 2.43, *SD* = 1.68, CI: 2.20, 2.67) than women (*M* = 1.95, *SD* = 1.42, CI: 1.72, 2.19).

For ratings of anger, an interaction between first emotion and reactive emotion emerged, *F*(2,334) = 4.04, *p* = 0.018, $\eta_{p}^{2}$ = 0.02. As can be seen in Table 3, anger expressions in response to anger, were perceived angrier than in any other condition. The next most intense rating was for anger expressions in response to sadness, which was higher than in any remaining condition. When sadness was responded to with fear, fear expressions were rated as angrier than when sadness was responded to with happiness. No other differences between conditions emerged.

**Table 3:** Perceived intensity of reactive emotions of anger and fear as a function of first expresser’s emotion and reactive emotion – Study 3.

	First emotion											
	
	Anger						Sadness					
	
	*Reactive emotion*											
	
	*Fear*		*Anger*		*Happiness*		Fear		*Anger*		*Happiness*	
						
Perceived emotion	*M SD*	95% CI	*M SD*	95% CI	*M SD*	95% CI	*M SD*	95% CI	*M SD*	95% CI	*M SD*	95% CI
Anger	2.25_cd_ 1.58	1.84, 2.66	5.16_a_ 1.36	4.74, 5.58	2.13_c_ 1.67	1.71, 2.53	2.81_d_ 1.67	2.38, 3.26	4.52_b_ 1.90	4.09, 4.93	2.04_c_ 1.47	1.63, 2.49
Fear	5.03_a_ 1.80	4.61, 5.45	3.12_c_ 1.68	2.67, 3.53	2.15_d_ 1.48	1.70, 2.54	4.15_b_ 1.79	3.71, 4.60	3.33_c_ 1.76	2.90, 3.76	1.82_d_ 1.34	1.38, 2.27


*Means with different subscripts differ at *p* < 0.05.*

A significant interaction between first emotion and reactive emotion also emerged for fear ratings, *F*(2,334) = 3.15, *p* = 0.04, $\eta_{p}^{2}$ = 0.02, as the lower part of Table 3 indicates, in response to anger, fear expressions were rated as more fearful than in any other condition, followed by the condition were fear was a response to sadness, which was still higher than in any other condition. Anger expressions in response to sadness or to anger, which did not differ, were rated as more fearful than happiness expressions in response to sadness or to anger which did not differ. Thus, overall, the focal emotion for each reactive emotion were perceived as planned; yet, as expected, expressions were rated as less intense and more mixed than the more intense expressions used in Study 1 and 2.

#### Perceived social power of first expresser

First, a 2 (First expression) × 2 (Gender of expressers) × 4 (Reactive emotion) ANOVA was conducted on ratings of social power. A significant main effect of first emotion emerged, *F*(1,441) = 197.44, *p* < 0.001, $\eta_{p}^{2}$ = 0.31, such that individuals who showed anger were rated as higher in power (*M* = 4.87, *SD* = 0.83, CI: 4.73, 4.98) than those who showed sadness (*M* = 3.60, *SD* = 1.22, CI: 3.45, 3.71). A significant main effect of second emotion, *F*(3,441) = 14.43, *p* < 0.001, $\eta_{p}^{2}$ = 0.09, was qualified by a first emotion by second emotion interaction *F*(3,441) = 8.05, *p* < 0.001, $\eta_{p}^{2}$ = 0.05. As shown in Table 2, compared to sadness shown alone, all reactive emotions increased perceptions of social power of the sad person. The increase was highest for anger followed by fear and significantly lower for happiness.

Compared to anger shown alone, anger reacted to with fear lead to increased attributions of social power. No other reactive emotion led to significantly different attributions when compared to anger alone.

Only the effect of fear responses to both angry and sad expressers replicated previous findings. It is curious that both happiness and anger when shown in response to sadness increased social power. In fact, we had expected that these expressions would either not impact on the power attributed to a sad person or reduce it. Also we had expected that anger and happiness responses to angry expressions would reduce attributions of social power to an angry expresser, which was not found.

The MR model for attributions of power to sad expressions explained 13% of the variance, *F*(4,161) = 6.07, *p* < 0.001. Only reactive emotion ratings of fear significantly and positively predicted attributions of social power (β = 0.25, *p* = 0.002, CI: 0.09, 0.40). The MR model for attributions of power to anger expressions explained 12% of the variance, *F*(4,175) = 5.88, *p* < 0.001. Significant effects emerged for anger (β = -0.23, *p* = 0.005, CI: -0.41, -0.07) and happiness (β = -0.23, *p* = 0.016, CI: -0.41, -0.04) which both decreased attributed power. Thus, the regressions based on the actual ratings of the expressions yielded a different picture than the ANOVA comparing reactive emotion conditions with the ratings for the expression shown alone. The findings from the MR are also more congruent with findings from Study 2. The emotion ratings reported above, might give an insight into the reason for this. As intended, the emotions were more subtle and hence rated less intensely, but also less distinctly. Further, a stronger interaction between first and second emotion was observed. Hence, the categorical emotion conditions may not have reflected the actual perceived emotions as closely as was the case for Study 2. Thus, while we can say that reactive emotions did make a difference for the attribution of power when compared to judgments of the expression alone, the direction and intensity of the impact depend strongly on the actual emotion perceived rather than on categorical emotion conditions.

#### Perceived acceptance of power by the second person

We then assessed to what degree the reactive emotions were perceived as supporting the notion that the first person has high(er) power. The MR for sadness expressions explained 33% of the variance, *F*(4,161) = 20.07, *p* < 0.001. Both fear reactions (β = 0.52, *p* < 0.001, CI: 0.38, 0.66) and perceived neutrality (β = 0.16, *p* = 0.015, CI: 0.03, 0.29) predicted less acceptance of social power. The MR for anger expressions explained 24% of the variance, *F*(4,175) = 13.84, *p* < 0.001). Only reactions of fear (β = 0.51, *p* < 0.001, CI: 0.36, 0.65) were perceived as supporting the notion that the first person has high(er) power. The finding for fear replicates findings from Study 1 and 2, however, neutrality did not contribute to the acceptance of power for either study. Also, the previously found effect for happiness as eroding a claim of high social power did not emerge.

### Mediation Analysis

As for Study 2, we calculated two saturated path-models, one for each first emotion. For sadness expressions, significant indirect effects emerged for fear (β = 0.14, *p* = 0.005, CI: 0.07, 0.40) and neutrality (β = 0.04, *p* = 0.020, CI: 0.02, 0.33) such that to the degree that these emotions were seen as signaling that the first person has high(er) power, the sad expresser was rated as higher in social power. For anger expressions, only a significant positive indirect effect for fear emerged (β = 0.20, *p* < 0.001, CI: 0.10, 0.34).

## Discussion

Based on the mediation analysis we were able to replicate the finding that the fear reactions of the addressee of sadness or anger are interpreted as acceptance of the notion that the first person has (high)er social power and in turn this support leads to attributions of higher social power by the participants. The effect for happiness reactions, which decreased such attributions for anger in Study 2 and to some degree also in Study 1, could not be replicated. In addition, ratings of the neutrality of the emotional reaction of the addressee of a sad expression also positively predicted social power as mediated by acceptance of power.

The findings overall suggest that participants paid attention to the expressions of both interaction partners and based their judgment of the social power of the first person on the expressions of both. This even when the expressions were subtle and dynamically evolved and overlapped.

Yet, the incongruence between ANOVA results, which were based on the categorical label of the focal emotion expression and the regression analyses, which were based on the actual emotion ratings effectuated by the participants, suggest that the effects of subtle emotions, which are perceived as more mixed and less distinctive, can not necessarily be predicted by the focal emotion alone. This in turn points to the importance of secondary emotion ratings. This is also evident from the effect of neutrality observed here. In Studies 1 and 2 only neutral expressions (which were not included in Study 3) were rated as neutral. But the more subtle expressions used here were rated as somewhat neutral. One speculation could be that an expressive reaction that is seen as emotional but somewhat controlled or constrained and thus somewhat neutral is perceived as indicative of the social power of the person it is addressed to. This is an interesting question for future research as it suggests that efforts at emotion regulation could have social signal value in their own right.

## General Discussion

The congruent finding of all three studies suggests that reactive fear is a strong signal of the social power of another person. It also supports the notion that observers base judgments of social power not only on the expression of the person whose social power they judge but also on the reactions of their interaction partner. However, the effect of reactive emotions was clearest when prototypical expressions were shown as dynamic videos one after the other. This allowed participants to clearly see the expressions and facilitated their labeling. Once emotion expressions were more subtle and shown concurrently, only the effect of reactive fear remained stable. More importantly in Study 3 it became evident that not only the social signal value of the focal emotion (i.e., fear for a fear expression) but also the secondary emotions that can be perceived in such expressions (i.e., neutrality in a fear expression or fear in a happy expression) are relevant. This is an interesting finding as previous research on the attribution of social power based on facial expressions has not only focused exclusively on the expression of the person whose power is to be judged but also exclusively focused on focal emotions, completely neglecting secondary emotions.

That secondary emotions are of importance in social interactions has been shown in recent research that links the perception of secondary emotions to the perception of social interaction quality such that to the degree that people perceive more intense secondary emotions they report less satisfying social interactions (Hess et al., 2016b). If secondary emotions also interfere with the perception of social attributes such as power or affiliation, this could be one path to explain this reduced social interaction quality.

In sum, the results of three studies suggest that the emotional reactions of the addressee of emotion expressions are meaningful signals which are used to infer the social power of the sender of the first expression. As discussed above, social power can be best conceived of as a person’s ability to influence others (Keltner et al., 2003). Emotion’s expressions can serve as cues to this ability (Knutson, 1996; Tiedens, 2001; Hareli et al., 2009) but also as signals of power (Hareli and David, 2017; Scarantino, 2017). Accordingly, the way an interaction partner reacts to such expressions is important for the degree to which such a signal should be believed. Hence, observers should prevail themselves of this information and they do.

The results also suggest that this basic finding is not depended on the somewhat artificial approach chosen by Hareli and David (2017) and by us in Study 1 and to a lesser degree in Study 2. Overall, the use of a set of studies that gradually and in a controlled manner add to the complexity involved in the perception of emotions in a social interaction enabled us to carefully assess the factors that influence how reactive emotions contribute to social judgments of power. Taken together, our research shows that the social signal value of emotion expressions depends in part on the emotional reaction of the interaction partner. Thus, the social signal value of emotions does not stand alone but has to be understood in the fuller context of the interaction. The present research highlights the importance of studying the social signal value of emotions in an interactional context and to acknowledge that observers do not necessarily perceive emotions as “pure” instantiations of a single emotional state, but more often as mixed. This is especially the case for the more subtle dynamic emotion displays that are typical for real life interactions.

## Ethics Statement

This study was carried out in accordance with the ethical standards of the American Psychological Association and the ethics committee of the faculty of management of the University of Haifa. The protocol was approved by the ethics committee of the faculty of management of the University of Haifa. All subjects gave written informed consent in accordance with the Declaration of Helsinki.

## Author Contributions

SH planned the studies and supervised its running and contributed in writing the paper, and conducing the analyses. MH was running the studies, created the experiments, and helped with the writing and analysis. UH helped plan the studies, contributed in writing the paper, and was leading the analyses.

## Conflict of Interest Statement

The authors declare that the research was conducted in the absence of any commercial or financial relationships that could be construed as a potential conflict of interest.

## Footnotes

1 Since Cronbach’s alpha, as a measure of reliability of a composite measure is considered to rely on assumptions that are often violated, we also report Omega as a measure of Composite Reliability (McNeish, 2017). This was done for all constructs across the studies.

2 www.techsmith.com/camtasia.html

3 www.fantamorph.com
